# Broadening the View: Substance P and Its Metabolism in Pruritus‐Related Diseases

**DOI:** 10.1111/1346-8138.70328

**Published:** 2026-06-04

**Authors:** Thomas Walter, Bjoern B. Burckhardt

**Affiliations:** ^1^ Individualized Pharmacotherapy, Institute of Pharmaceutical and Medicinal Chemistry, University of Münster Münster Germany

**Keywords:** biased signaling, bioactive metabolites, neuroinflammatory diseases, pruritus, substance P

## Abstract

Chronic pruritus is a debilitating symptom accompanying numerous inflammatory skin diseases and remains a major therapeutic challenge. Neurogenic inflammation plays a central role in its pathogenesis, with the tachykinin substance P acting as a key mediator at the interface of the nervous system, immune cells, and cutaneous tissues. While substantial research has focused on substance P signaling via the neurokinin‐1 receptor, clinical trials targeting this pathway have yielded inconsistent results, suggesting an incomplete understanding of substance P‐mediated mechanisms in pruritic disorders. This review broadens the current perspective by highlighting the importance of substance P metabolism and the biological activity of its metabolites in pruritus‐related diseases. Many C‐terminal substance P metabolites retain neurokinin 1‐receptor affinity but induce biased intracellular signaling. In contrast, N‐terminal metabolites can activate alternative receptors such as mas‐related G protein‐coupled receptor X2, promoting mast cell degranulation, neurogenic inflammation, and itch, or exert counter‐regulatory effects via yet unidentified targets. Importantly, the expression and activity of substance P‐degrading proteases are altered in pruritic skin diseases, shifting the balance toward biologically active, pruritogenic metabolites. In conclusion, substance P, its metabolites, receptor isoforms, and metabolizing enzymes form a complex regulatory network that fine‐tunes itch, pain, and inflammatory signaling. A deeper understanding of this interplay may explain the limited efficacy of current therapeutic approaches and might offer novel targeted treatment strategies in chronic pruritus.

## 1 Introduction

Neurogenic inflammation has a key role in the pathogenesis of pain and pruritus [1]. Many inflammatory diseases of the skin are accompanied by an additional pruritic component [2]. The tachykinin substance P (SP) is an important mediator involved in neurogenic inflammation and participates in the peripheral sensitization to pruritogenic triggers [3, 4]. SP exerts its effects both in the central nervous system (CNS) and in peripheral tissue. Not only does it function as a neurotransmitter, but it further translates neuronal signals into a cellular response, stimulates cells in an autocrine manner, and participates in the complex crosstalk between immune cells [5, 6]. Various attempts to target the SP‐related signaling pathways in pruritus‐related diseases, mostly by inhibiting the neurokinin‐1 receptor (NK1R), did not meet primary endpoints in clinical studies or led to ambiguous results [7, 8, 9, 10]. This indicates limited understanding of the function of SP in pruritus‐related diseases.

In recent years, the understanding of the signaling of SP has broadened. The classical full‐length NK1R couples to at least two distinct G proteins, raising the option to different signaling cascades [11, 12]. Next to the classical full‐length NK1R (NK1R‐F), there is a truncated isoform of the receptor (NK1R‐T) which differs in the way and duration of signaling [13]. NK1R‐T predominates in the peripheral tissues, but is also found in the CNS, where the classical NK1R‐F is mainly expressed [14, 15]. Further, the mas‐related G protein‐coupled receptor X2 (MRGPRX2) could be identified as an additional place of action for SP and other tachykinins in peripheral tissues [16]. It is predominantly expressed in mast cells and leads to degranulation and subsequent release of histamine and tryptase, further aggravating inflammatory processes, activating fibroblasts and matrix metalloproteases, promoting fibrosis [17]. Aberrant expression of receptors and ion channels (e.g., TRPV1, MRGPRX2), tissue invasion and activity of immune cells and changes in innervation density in the periphery lead to peripheral sensitization to pruritogenic triggers, while changes in the itch‐related brain areas in the CNS lead to central sensitization [4].

Additionally, many of the possible resulting SP metabolites retain receptor affinity and activity, extending the duration of action, but also altering intracellular signaling cascades towards a biased signaling [12, 18]. Degradation of SP is tissue specific and depends on the expressed proteases. Pathophysiological processes alter the tissue environment and influence protease activity and thus the metabolism of bioactive peptides such as SP. Depending on the cleaving site of the predominant proteases, receptor activity may be retained, diminished, or eliminated [12, 18].

Therefore, this review provides an overview of the physiological and pathophysiological implications of prolonged and shortened forms of SP with special attention to pruritus‐associated diseases. Emphasis is given to its metabolism in disease state and the biased signaling of the active SP metabolites.

## 2 Substance P Biosynthesis

**FIGURE 1:**
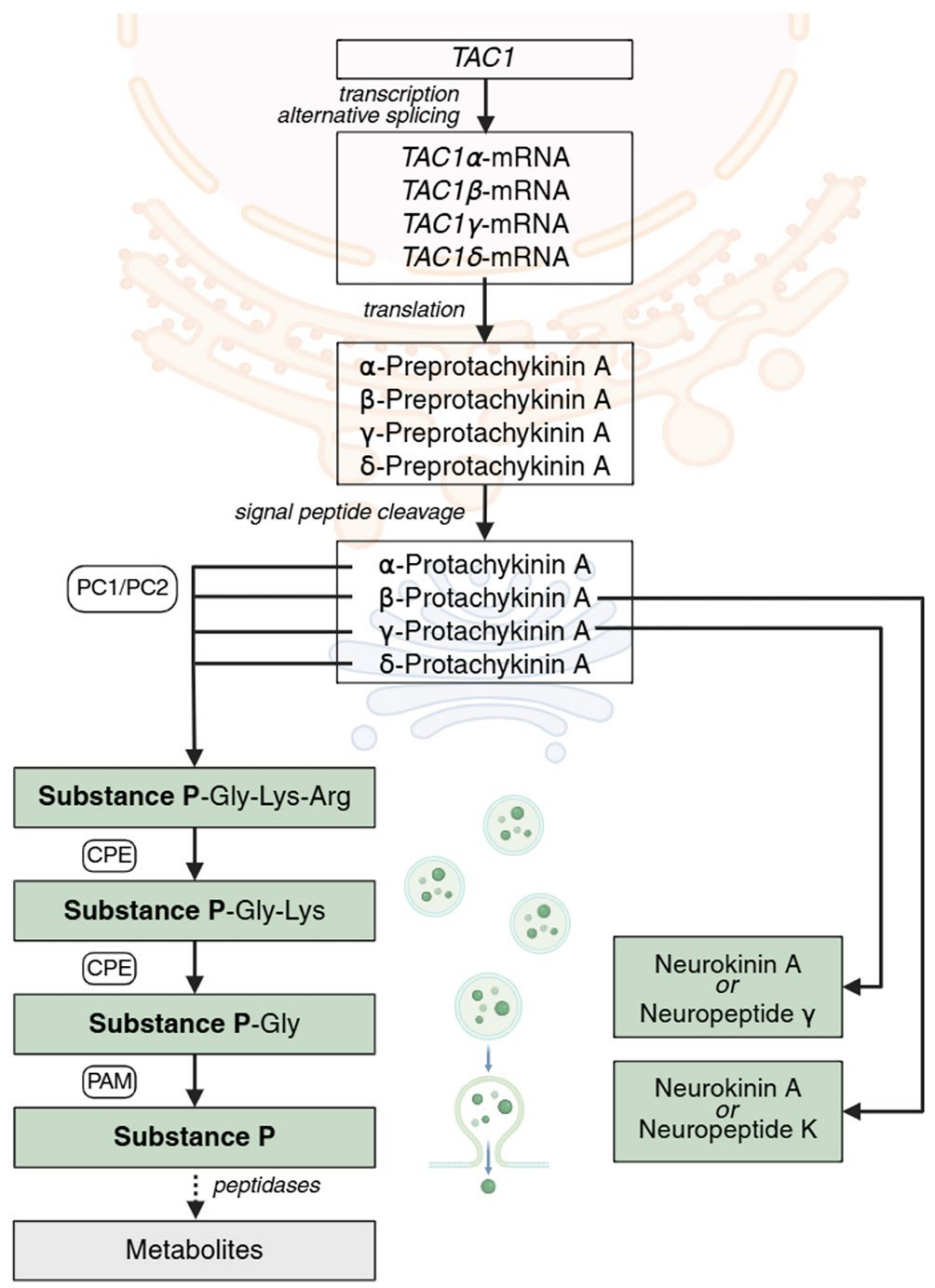
Substance P biosynthesis. Transcription of the TAC1 gene and alternative splicing leads to four TAC1 mRNA variants, which are translated into α‐, β‐, γ‐, and δ‐Preprotachykinin A, respectively. The cleavage of the signal peptide during co‐translational translocation into the endoplasmic reticulum results in Protachykinin A. Substance P is included in each Protachykinin A, while Neurokinin A is only included in β‐ and γ‐Protachykinin A. The N‐terminal prolonged peptides of Neurokinin A, Neuropeptide K and Neuropeptide γ, are included in β‐ and γ‐Protachykinin A, respectively. Each tachykinin is cleaved with a ‐Gly‐Lys‐Arg sequence on its C‐terminal end, which is further processed to the final peptide with an amidated methionine at the C‐terminus. CPE, Carboxypeptidase E; PAM, Peptidyl Glycyl Amidase; PC, Prohormone Convertase. Created with BioRender.com.

The peptide SP is encoded on the *TAC1* gene located on chromosome 7. The *TAC1* gene and the transcribed peptides are highly conserved among all mammals, underscoring their fundamental physiological significance [19]. Alternative splicing of the transcribed pre‐mRNA generates four distinct variants through inclusion or exclusion of the exons 4 and 6, namely *TAC1α*‐, *TAC1β*‐, *TAC1γ*‐, and *TAC1δ*‐mRNA [20, 21, 22]. Although all splice variants can be found in extracts from the murine jugular‐nodose ganglion complex, individual neurons express only a single splice variant, indicating that each neuron exhibits a distinct tachykinergic phenotype [23]. Translation of the four splicing variants leads to the precursor protein α‐, β‐, γ‐, or δ‐Preprotachykinin A (PPTA). SP is included in each PPTA, as shown in Figure 1.

In cultured human fibroblasts and keratinocytes, exogenous SP induces β‐PPTA‐mRNA expression and leads to a subsequent increase of SP peptide, indicating an autocrine mechanism [24, 25]. Additionally, many of the PPTA‐positive neurons responded to noxious and pruritic stimuli, especially histamine induced itch [26]. Further processing of PPTA leads to the direct intermediates that are C‐terminally extended with the sequence ‐Gly‐Lys‐Arg (e.g., SP‐Gly‐Lys‐Arg) and sequentially cleaved to full‐length SP (Figure 1) [27, 28]. While biological activity of these direct intermediates at the NK1R is unlikely due to the C‐terminal modification, activity at the MRGPRX2 remains theoretically possible, since binding here occurs with the N‐terminal, polycationic end [29]. However, their role in pruritus‐related diseases has not been reported yet.

## 3 Substance P and Its Function in Pruritus‐Related Diseases

SP is the most well‐known tachykinin and broadly expressed in the human CNS, peripheral nerves, immune cells and tissues [3, 24, 30, 31, 32]. It transmits pain and itch signals via primary afferent neurons to the dorsal root ganglion and acts there as a neurotransmitter [3, 4]. There are three distinct tachykinin receptors, namely NK1R, NK2R and NK3R, of which SP has the highest affinity for NK1R [3]. The receptor exists in two isoforms, NK1R‐F with 407 amino acids and the C‐terminally truncated NK1R‐T with 311 amino acids. The isoforms differ in signaling, in form of missing calcium mobilization, no NF‐κB activation and a delayed response in ERK1/2 activation for NK1R‐T and approximately ten times higher concentrations of SP are required for activation [33]. The NK1R‐F binds to β‐arrestin 2 and recruits MAPK/ERK kinase kinase (MEKK), extracellular signal‐regulated kinase 1/2 (ERK1/2) and Src after internalization, forming an endosomal signalosome [3]. The missing intracellular C‐terminal tail in NK1R‐T prevents β‐arrestin 2 binding to NK1R‐T, hindering de‐sensitization and endosomal signaling [3, 14]. Therefore, signaling on the NK1R‐T is delayed but prolonged compared to NK1R‐F. While the NK1R‐F can either be Gq‐ or Gs‐protein coupled, an in vitro study could not demonstrate G‐protein dissociation after activation of NK1R‐T [12, 34]. This suggests that the NK1R‐T might have other signaling pathways. In most tissues where NK1R is expressed, both isoforms can be found, with a higher prevalence of NK1R‐F in the CNS and NK1R‐T in peripheral tissue [15]. NK1R‐T is further expressed on several immune cells like monocytes, NK cells and T cells [14, 35]. Taken together, the need for high SP concentrations and altered signaling suggests that the NK1R‐T acts as a fine‐tuning instrument for SP signaling in the immune system and peripheral tissue [14].

**FIGURE 2:**
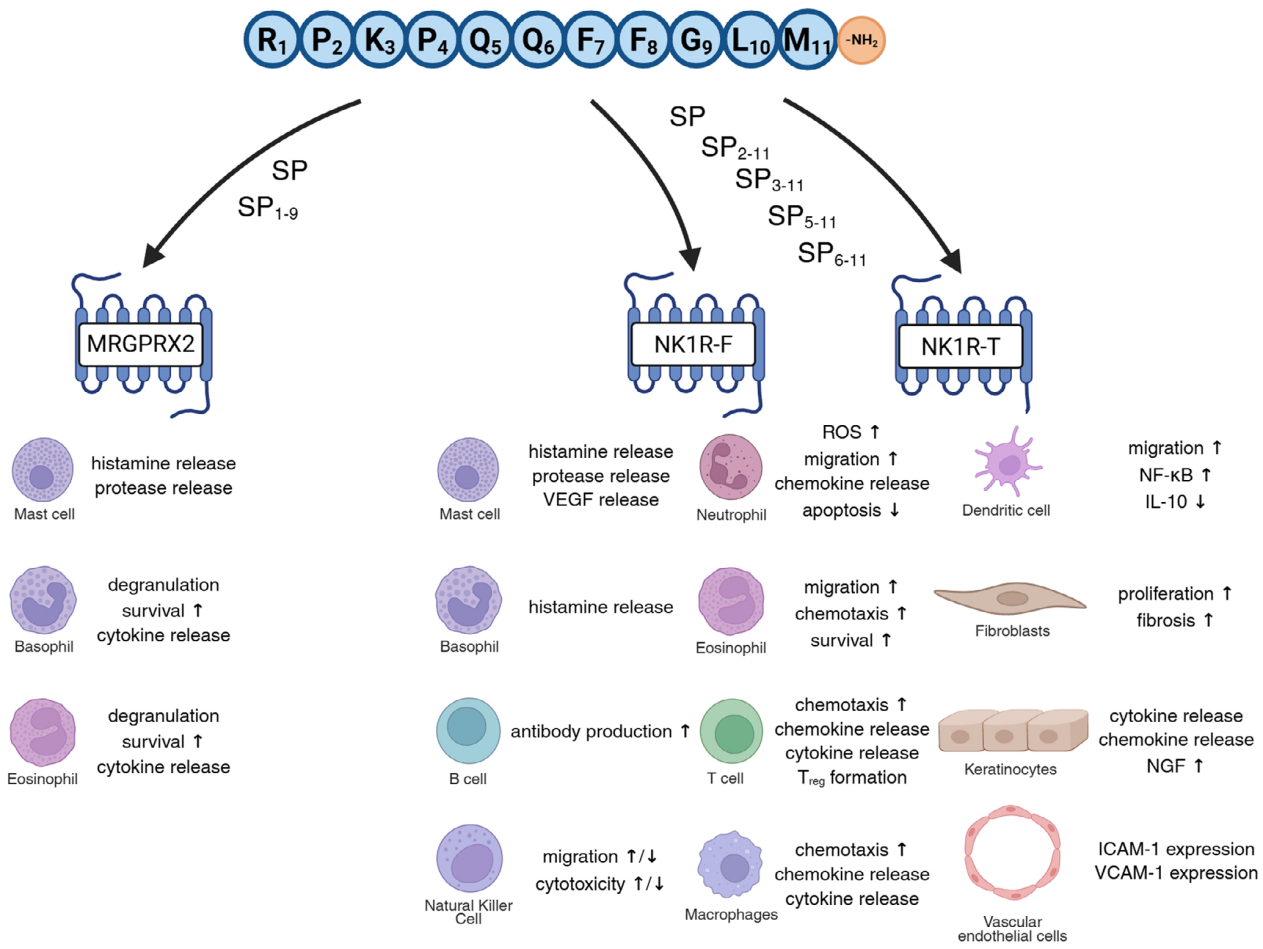
Resulting effects of substance P and metabolites on skin and immune cells. The substance P peptide sequence is depicted in the single letter amino acid notation. ICAM, Intercellular Adhesion Molecule; IL, Interleukin; MRGPRX2, Mas‐Related G Protein Coupled Receptor X2; NF‐κB, Nuclear Factor kappa‐light‐chain‐enhancer of activated B cells; NGF, Nerve Growth Factor; NK1R, Neurokinin 1 Receptor; ROS, Reactive Oxygen Species; SP, Substance P; VCAM, Vascular Cell Adhesion Molecule; VEGF, vascular endothelial growth factor. Created with BioRender.com.

Nociceptive stimuli lead to the release of SP from neurons into the surrounding tissue, where it elicits arteriolar dilation, plasma extravasation, and granulocyte infiltration (neurogenic inflammation) [3, 5, 36]. SP‐NK1R interaction induces NF‐κB and, in turn, leads to the production of proinflammatory cytokines interleukin‐1 (IL‐1), IL‐6, tumor necrosis factor alpha (TNF‐α), macrophage inflammatory protein‐1 beta (MIP‐1β), and interferon gamma (IFNγ) [37]. SP induces the expression of intercellular adhesion molecule‐1 (ICAM‐1) and endothelial leukocyte adhesion molecule‐1 (ELAM‐1), promoting neutrophil migration via NK1R [38, 39, 40, 41]. It can activate eosinophils and induce degranulation on mast cells, which, in turn, leads to the release of pro‐inflammatory cytokines and chemokines [16, 42]. Further effects of SP are depicted in Figure 2.

In addition to the inflammatory response, the NK1R plays a crucial role in transmitting pruritus signals. It is found in dorsal horn neurons and mediates acute and chronic itch [43, 44]. Therefore, regarding the pruritic signaling, the NK1R is important for release of directly pruritogenic mediators in the skin and for the processing of itch signals in peripheral and spinal neurons [45]. A differentiation of NK1R‐F and NK1R‐T regarding itch signaling has not been reported yet.

In addition to NK1R, SP is an agonist for MRGPRX2, which is expressed on mast cells and eosinophil and basophil granulocytes [16, 46]. Upon activation it leads to FcεR1‐independent degranulation, release of histamine and subsequently an itch sensation. Mast cell degranulation also releases chymase and tryptase, which in turn leads to activation of fibroblasts and matrix metalloproteases and subsequently to fibrosis [47]. The reactivity of mast cells to SP seems to be specific for the anatomical site, with high affinity on skin derived mast cells [48]. Many effects that were assigned to SP‐NK1R interaction, especially for mast cell‐mediated neurogenic inflammation, pain and itch, are likely to have a SP‐MRGPRX2 component [16, 49]. High SP concentrations are required for MRGPRX2 activation, but these can be achieved in the immediate tissue environment, where mast cells lie in direct proximity to nerve terminals [50, 51, 52]. As recently reviewed, MRGPRX2 plays a role in the pathogenesis of atopic dermatitis, psoriasis, chronic spontaneous urticaria, chronic pruritus and rosacea, either determined by elevation of typical MRGPRX2 agonists (e.g., SP, vasoactive intestinal peptide, LL37, cortistatin‐14) or by elevated levels of MRGPRX2 [53]. Next to the potential as a serum biomarker in chronic spontaneous urticaria, MRGPRX2 expression is also elevated in skin‐derived mast cells [54, 55]. The expression of MRGPRX2 on DRG neurons has been reported in several studies utilizing immunohistochemistry (IHC) and quantitative polymerase chain reaction (qPCR) and real time PCR, but is under discussion in the light of recent RNAseq data [56, 57, 58, 59]. A direct action of SP as a pruritogen via MRGPRX2 on pruriceptors has been shown in vitro [49]. Further, an indirect pruritic pathway of SP via MRGPRX2 is initiated via mast cell degradation, since the released mediators and proteases can induce itch.

Effects of SP on eosinophil and basophil granulocytes implicate enhanced migration, survival and degranulation [42, 60]. While the NK1R has a low expression on eosinophils, MRGPRX2 is expressed on basophil and eosinophil granulocytes [42, 46]. However, an SP‐MRGPRX2 interaction on eosinophils and possible effects have not yet been tested.

In the skin, SP is found in primary sensory nerves and the NK1R is expressed and self‐induced in human keratinocytes and fibroblasts [61, 62]. SP immunoreactivity is predominantly detected on the dermo‐epidermal junction, where it mediates pain and itch via small‐diameter C‐fibers [63, 64]. The autocrine mechanism of SP leads to aggravation of the inflammatory process, while later on the expression of neprilysin (NEP) is induced, promoting the proteolytic degradation of the tachykinin [24]. Moreover, the SP‐NK1R interaction on keratinocytes leads to secretion of inflammatory cytokines and chemokines, the production of nerve growth factor, and the NK1R and SP expression is increased in an autocrine manner [65, 66].

The plasma level of SP is elevated in **atopic dermatitis** and correlates significantly with disease severity and the likewise elevated nerve growth factor [67, 68]. Significantly elevated *TAC1* and *TACR1* gene expression compared to healthy skin was shown by transcriptome analysis of skin biopsies from lesional skin in patients with atopic dermatitis and psoriasis [64]. A significantly elevated number of SP‐positive nerve fibers on the dermo‐epidermal junction and significantly overexpressed NK1R in the lesional epidermis of atopic dermatitis and psoriasis compared to non‐lesional skin (NK1R expression in lesional compared to non‐lesional skin in atopic dermatitis +88%; *p* < 0.0001 and in psoriasis +30.1%; *p* < 0.01) confirmed the transcriptome results [64].

The involvement of neuroinflammatory components in **psoriasis** has been postulated, when nerve injury or denervation of the psoriasis‐affected area showed improvement of skin lesions. Depletion of neuropeptides (e.g., SP, VIP, CGRP) has been suggested as a potential mechanism [69]. SP mediates neutrophil chemotaxis, mitogenicity for connective tissue and epithelial cells, T‐cell activation, and stimulates IL‐1 secretion from keratinocytes in psoriasis [63].

In **prurigo nodularis** and **chronic pruritus**, the density of SP‐positive nerve fibers is significantly increased in lesional skin compared to non‐lesional skin [70]. The NK1R expression is higher in keratinocytes in prurigo nodularis patients, and treatment with the antagonist aprepitant showed a significant reduction in pruritus intensity in 12 patients with prurigo nodularis (pruritus on visual analogue scale before treatment 6.3 ± 1.24, after 4‐week treatment period 4.5 ± 2.8, *p* = 0.02; mean ± standard deviation) [71]. In contrast, the phase‐2 trial APREPRU (Eudra‐CT 2013‐001601‐85) failed to confirm the beneficial effects of aprepitant in prurigo nodularis [72]. Another NK1R antagonist, serlopitant, showed a significant reduction of pruritus intensity in chronic pruritus in a phase‐2 trial (NCT01951274) but failed to meet the primary endpoints in two phase‐3 trials (NCT03546816; NCT03677401) [73].

Ambiguous results for serum SP in **chronic spontaneous urticaria** have been reported. While initial reports found no significant changes in serum SP, later studies found significant changes between healthy controls and severe chronic spontaneous urticaria [54, 74, 75]. The number of basophils and the percentage of SP‐positive and NK1R‐positive basophils were elevated in the blood of chronic spontaneous urticaria patients compared to healthy control, and challenging the isolated basophils with SP led to a more pronounced degranulation and histamine release [76].

The level of SP and the number of SP‐positive and NK1R‐positive immune cells is elevated in the plasma of **eczema** patients, while the number of SP‐positive and NK1R‐positive immune cells is also elevated in human eczema skin [41].

In **bullous pemphigoid**, the number of SP‐positive cells is elevated in skin lesions and correlates with itch severity measured with the visual analogue scale (*r* = 0.548, *p* = 0.002), while there is also a weak correlation for NK1R‐positive cells with itch severity (*r* = 0.422, *p* = 0.025) [77].

SP is significantly elevated in **mycosis fungoides**, a common type auf cutaneous T cell lymphoma, compared to healthy control (*p* < 0.001). Additionally, the serum concentrations were significantly elevated in acute pruritic versus non‐pruritic mycosis fungoides (*p* = 0.040) [78]. The use of aprepitant for refractory pruritus in **cutaneous T cell lymphoma** has been effective in several cases and case studies using a standard dosing regimen, indicating the involvement of NK1R in pruritus [79]. An additional MRGPRX2 component has been proposed for mycosis fungoides, since the number of MRGPRX2‐positive mast cells is increased in lesional skin compared to non‐lesional skin and healthy control (15.12 vs. 6.84 vs. 5.51 cells/mm^2^; *p* = 0.04) [80].

Pruritus is a common symptom for **allergic contact dermatitis** and a key factor influencing the quality of life of patients [81]. SP is increased in lesional skin of patients with allergic contact dermatitis and neurogenic inflammation is mediated by SP‐NK1R interaction [82, 83, 84]. Likewise, the expression of NK1R is elevated on mRNA and protein levels in lesional skin of atopic dermatitis patients [82].

Efficacy of NK1R‐antagonists in animal models and the subsequent failure in the following clinical studies might be explained by an off‐target activity of aprepitant on the mouse MrgprB2, but not on the human homologue MRGPRX2 [85]. Therefore, SP‐mediated inflammation through interaction with the MRGPRs is inhibited in the mouse model, but not in the human trials. Nevertheless, the NK1R‐antagonists such as aprepitant are still suggested for refractory chronic pruritus in the European S2k guideline on chronic pruritus [86, 87]. The use of NK1R antagonists in pruritus has recently been discussed in a systematic review and meta‐analysis [7]. Although there seems to be a promising therapeutic potential targeting MRGPRX2, there are currently no treatment options available [17]. The small molecule EP262 was terminated in Phase 2 (NCT06077773). For EVO756, the recruiting process for Phase 2b studies for atopic dermatitis (NCT07150845) and chronic spontaneous urticaria (NCT06873516) is currently running.

## 4 Substance P Metabolites and Their Function in Pruritus‐Related Diseases

The biological activity of SP is curtailed by degradation mediated by a diverse array of peptidases, leading to the formation of multiple metabolites. While cleavage is possible at virtually any peptide bond, only a limited subset of these potential metabolites has been detected in vivo. The spectrum of observed metabolites differs among plasma, the CNS, and peripheral tissues, reflecting the distinct enzymatic environments that govern SP turnover in each compartment.

**FIGURE 3:**
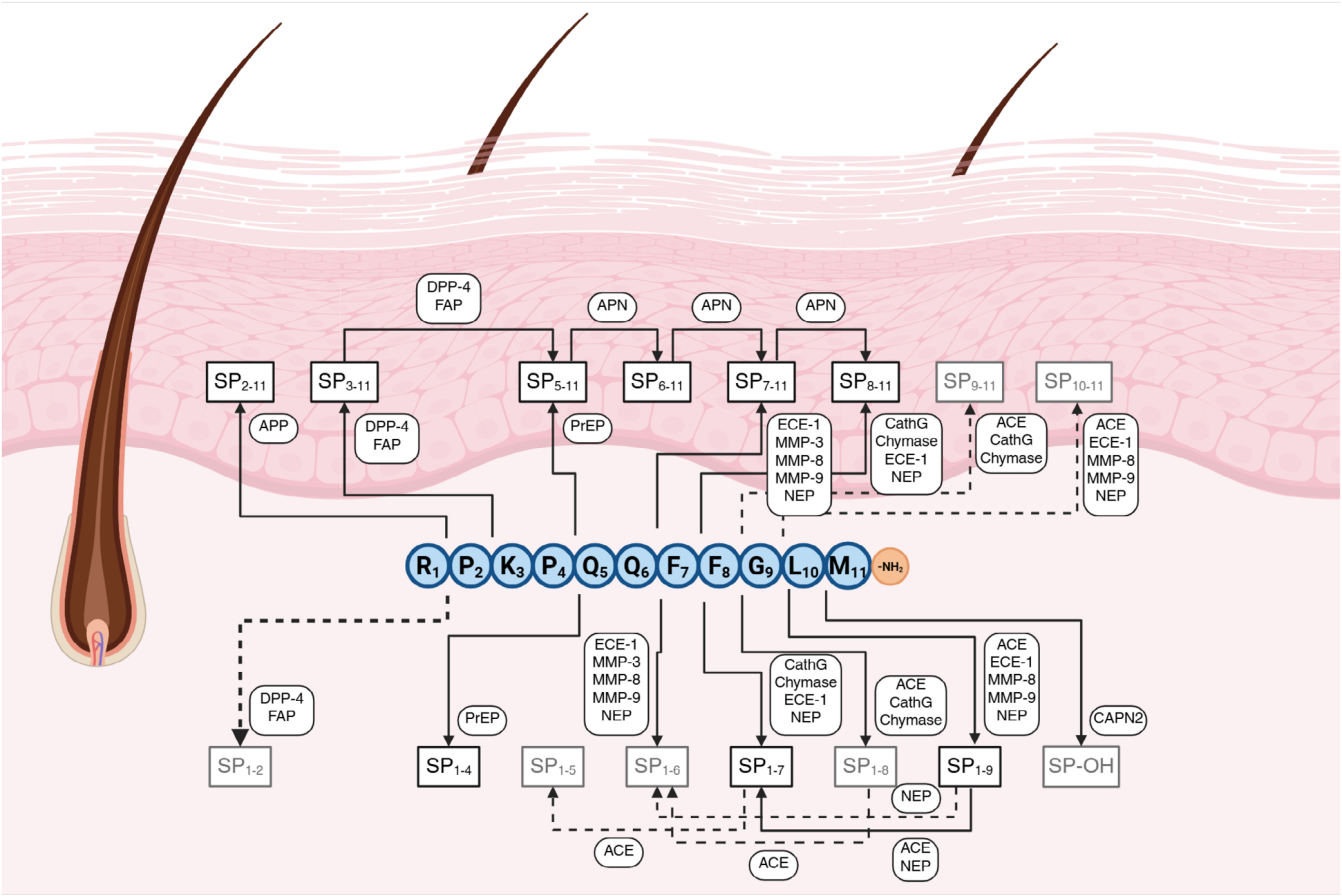
Overview of the possible degradation pathways of substance P in the skin. Confirmed degradation pathways are depicted in black with solid lines, while theoretical degradation pathways are depicted in gray and with dashed lines. The substance P peptide sequence is shown in the single letter amino acid notation. ACE, Angiotensin Converting Enzyme; APN, Aminopeptidase N; APP, Aminopeptidase P; CathG, Cathepsin G; CAPN, Calpain; DPP, Dipeptidylpeptidase; ECE, Endothelin Converting Enzyme; FAP, Fibroblast Activation Protein alpha; MMP, Matrix Metalloprotease; NEP, Neprilysin; PrEP, Prolyl Endopeptidase; SP, Substance P. Created with BioRender.com.

Specifically in the skin, in vivo data on SP metabolism is limited. Considering the presence of SP‐degrading peptidases in the skin and skin‐related cells, a hypothetical degradation pattern can be designed (Figure 3, Table 1). The main degrading enzyme for SP in tissue is NEP [120]. SP and SP_1‐7_ can increase the mRNA expression of NEP, indicating that SP and its metabolite induce its own degradation [25, 126]. Endothelin‐converting enzyme‐1 degrades SP in the internalized SP‐NK1R‐β‐arrestin complex, limiting the signalosome activity via β‐arrestin‐MAPK‐ERK2 and promoting resensitization of NK1R [127, 128]. The resulting fragments (SP_7‐11_, SP_8‐11_, SP_10‐11_) have low to none receptor affinity on NK1R [12]. Aminopeptidase P ‐like activity in the human dermis has been indirectly proven by potentiating the bradykinin‐induced wheal response utilizing the inhibitor apstatin [98]. However, the resulting metabolite SP_2‐11_ is seldom detected in vitro and in vivo [99]. Aminopeptidase N is not involved in primary SP metabolism, but may degrade SP_5‐11_ to SP_8‐11_ by sequentially cleaving single amino acids from the N‐terminus, therefore limiting the remaining activity of the metabolites [94, 95, 96]. Metabolization of SP by angiotensin‐converting enzyme (ACE) leads to loss of activity on NK1R [121]. Pruritus, urticaria, and psoriasis are common adverse effects of ACE inhibitor usage [129, 130]. Calpain 2 is capable of deamidating the C‐terminal amide of SP and is present in keratinocytes, dermal fibroblasts and vascular endothelial cells [101, 102]. The resulting free acid of SP, SP‐OH, is inactive on the NK1R [12]. The prolyl endopeptidase cleaves SP to the metabolites SP_1‐4_ and SP_5‐11_ and is highly expressed in the stratum granulosum, on sebaceous glands and hair follicles. Moderate expression is described in the stratum spinosum, basal cells and some immune cells like monocytes and basophils [124]. Some matrix metalloproteases (MMP) present in the skin can degrade SP. While MMP‐3 only has one cleavage site between Gln6‐Phe7, MMP‐8 and MMP‐9 additionally cleave between Gly9‐Leu10 [117, 118, 119]. The generated metabolite SP_1‐9_ remains active on the MRGPRX2 [18]. The neutrophile‐derived enzyme cathepsin G and the mast cell‐derived chymase both cleave the metabolites SP_1‐7_, SP_1‐8_ and SP_8‐11_, which have no activity on NK1R or MRGPRX2 [12, 18, 103].

**TABLE 1:** The proteases involved in SP degradation in skin and skin related cells.

Enzyme	EC	Cleavage site	Substance P metabolites			Location in the skin	References
			Primary		Secondary		
ACE	3.4.15.1	Phe8‐Gly9 Gly9‐Leu10	1–8 1–9 9–11 10–11	➔ ➔	1–6 1–7 ➔ 1–5	Fibroblasts Keratinocytes Vascular endothelial cells	[88, 89, 90, 91, 92, 93]
APN	3.4.11.2	Gln5‐Gln6 (only in 5‐11) Gln6‐Phe7 (only in 6‐11)	/		5–11 ➔ 6–11 ➔ 7–11	Fibroblasts Keratinocytes Dendritic cells Monocytes neutrophils	[94, 95, 96, 97]
APP	3.4.11.9	Arg1‐Pro2	2–11			B cells T cells Monocytes Human skin microvasculature	[98, 99, 100]
CAPN2	3.4.22.53	Met11‐NH2	SP‐OH			Keratinocytes Dermal fibroblasts Vascular endothelial cells	[101, 102]
Cathepsin G	3.4.21.20	Phe7‐Phe8 Phe8‐Gly9	1–7 1–8 8–11 9–11			Human Neutrophils mast cells	[103]
Chymase	3.4.21.39	Phe7‐Phe8 Phe8‐Gly9	1–7 1–8 8–11 9–11			Mast cell granules	[103]
DPP4	3.4.14.5	Pro2‐Lys3 Pro4‐Gln5 (only in 3‐11)	1–2 3–11	➔	5–11 1–9 ➔ 3–9 ➔ 5–9 1–7 ➔ 3–7 ➔ 5–7	Fibroblasts Keratinocytes Mast cells T cells Melanocytes Human sebocytes	[104, 105, 106, 107]
ECE‐1	3.4.24.71	Gln6‐Phe7 Phe7‐Phe8 Gly9‐Leu10	1–6 1–7 1–9 7–11 8–11 10–11			Epidermis Sebaceous glands Vascular endothelial cells	[108, 109, 110, 111, 112, 113]
FAP	3.4.21.B28	Pro2‐Lys3 Pro4‐Gln5 (only in 3‐11)	1–2 3–11	➔	5–11	Fibroblasts Melanocytes	[114, 115, 116]
MMP‐3	3.4.24.17	Gln6‐Phe7	1–6 7–11			Keratinocytes Dermal fibroblasts	[117, 118]
MMP‐8	3.4.24.34	Gln6‐Phe7 Gly9‐Leu10	1–6 1–9 7–11 10–11			PMN leukocytes Neutrophils	[117]
MMP‐9	3.4.24.35	Gln6‐Phe7 Gly9‐Leu10	1–6 1–9 7–11 10–11	➔	7–9	PMN leukocytes Keratinocytes	[117, 119]
NEP	3.4.24.11	Gln6‐Phe7 Phe7‐Phe8 Gly9‐Leu10	1–6 1–7 1–9 7–11 8–11 10–11	➔ ➔	7–9 8–9	Fibroblasts Keratinocytes Vascular endothelial cells	[89, 91, 92, 120, 121, 122, 123]
PrEP	3.4.21.26	Pro4‐Gln5	1–4 5–11			Basophils Monocytes Epidermis Sebaceous glands hair follicles	[124, 125]

Pruritus‐associated diseases alter the expression of several SP‐degrading peptidases. A decreased expression of NEP has been reported for the epidermal basal layer and the endothelia of blood vessels in itchy, lesional skin of psoriasis patients, leading to prolonged neuropeptide actions [131]. Chymase released through degranulation of mast cells, e.g., after SP‐MRGPRX2 interaction, seems to be reduced in psoriasis and AD, while the co‐released tryptase remains active. The consequence is a SP‐favored microenvironment [50].

Contrarily to the decreased activity of NEP and chymase, dipeptidylpeptidase‐4 (DPP‐4) is expressed in keratinocytes, fibroblasts, mast cells and CD4^+^ and CD8^+^ T cells in psoriatic lesions and its activity and mRNA levels are increased in psoriasis [104]. Soluble DPP‐4 plasma level is elevated in atopic dermatitis and correlates with eruption area and the activity of the closely related fibroblast activation protein alpha is elevated in chronically inflamed baboon skin [105, 114]. In psoriasis, aminopeptidase N is overexpressed in dermal fibroblasts in lesional and non‐lesional skin [97]. This results in a slower deactivating metabolism with shorter metabolites via NEP and chymase and promotes a metabolism towards C‐terminal metabolites that maintain activity at the NK1R (e.g., SP_3‐11_ and SP_5‐11_ by DPP‐4).

Expression of ACE is increased in allergic contact dermatitis and relevant for the limitation of disease progression [82, 84, 132]. For NEP, there are contradicting results regarding the pathophysiological alteration [84, 133]. Elevated protease expression is a key feature in pemphigoid diseases, and the SP‐degrading proteases cathepsin G, chymase, MMP‐3, and MMP‐9 are implicated in the pathology of bullous pemphigoid [134]. Nevertheless, the SP metabolism or metabolites of SP have not yet been studied in bullous pemphigoid. Despite this, it is noteworthy that the measured SP levels are significantly increased in a micromilieu of upregulated SP‐degrading proteases.

C‐terminal metabolites like SP_2‐11_, SP _3–11_, SP_5‐11_, and SP_6‐11_ retain activity at NK1R‐F but display biased signaling with decreasing peptide length. While the Gq‐mediated increase in intracellular calcium is more promiscuous to N‐terminal truncated metabolites and deamidation of the C‐terminus, the Gs‐mediated increase in cAMP is stepwise reduced with each cleaved N‐terminal amino acid and is absent after deamidation to SP‐free acid [12, 135]. Therefore, full‐length SP, SP_2‐11_, and SP_3‐11_ lead to a calcium and cAMP response in HEK293 cells, but the shorter metabolites SP_5‐11_ and SP_6‐11_ only increase intracellular calcium, but not cAMP [12]. SP_7‐11_ has a diminished response in the rise of intracellular calcium, and shorter metabolites are not active at the NK1R‐F [12]. A similar gradual decrease of Gs‐signaling with preserved Gq‐signaling could be demonstrated for the stepwise truncated fragments of human hemokinin‐1 on the NK1R [136]. For both, SP and human hemokinin‐1, the last six C‐terminal amino acids are the minimal length to activate NK1R [135, 136]. In contrast, recent experiments in COS7 cells displayed a simultaneous decrease for both signaling pathways [137]. This discrepancy can be explained by the different cell lines, with HEK293 cells being closer to human tissue. The publications concur that SP_6‐11_ is the shortest metabolite with relevant biological activity [135, 137].

Scratching behavior in mice increased after application of the metabolite SP_5‐11_, even exceeding the effect of full‐length SP [138]. Administration of a DPP‐4 inhibitor or ultraviolet B therapy could reduce the serum levels of SP_5‐11_ and scratching behavior [138, 139]. While the activity of the parent peptide is limited by proteases, an active uptake mechanism has been described for its metabolite SP_5‐11_ in the CNS [140, 141].

The C‐terminal end of SP is critical for receptor affinity and activity, and the N‐terminal metabolites have no residual activity on the NK1R. Instead, the N‐terminal metabolite SP_1‐9_ has affinity for MRGPRX2, where it binds via the polycationic N‐terminal end and leads to mast cell degranulation [18, 29, 47].

Anti‐allodynic, anti‐nociceptive and anti‐hyperalgesic effects are mediated by the N‐terminal metabolite SP_1‐7_ [142, 143, 144]. It remains biologically active, with many effects opposite to the parent peptide. Specifically, SP_1‐7_ exerts anxiolytic and analgesic effects that are not elicited by NK1R‐F, NK1R‐T or MRGPRX2 [144, 145, 146, 147]. Distinct binding sites for SP_1‐7_ could be detected in rat spinal cord membranes [148, 149, 150]. Since the anti‐nociceptive effect of SP_1‐7_ could be reversed by naloxone and (+)‐pentazocine, an implication of the sigma 1 receptor (S1R) has been suggested [144, 145, 151]. This ligand‐sensitive intracellular chaperone regulates the expression or function of a multitude of receptors, ion channels and kinases either on the plasma membrane, in the cytosol, on the nuclear envelope or the endoplasmic reticulum‐mitochondrion interface [152]. The anti‐nociceptive, anti‐allodynic and anti‐hyperalgesic effects mediated by inhibition of the sigma receptor system are promising for future therapeutic use [145, 147, 153]. Whether SP_1‐7_ or designed peptidomimetics also yield antipruritic effects like some S1R antagonists (e.g., E153) needs to be elucidated [154].

## 5 Conclusion

Substance P is a central driver of neurogenic inflammation and pruritus, yet its role extends far beyond signaling by the full‐length peptide at the neurokinin‐1 receptor. This review highlights that tissue‐specific metabolism of substance P generates a diverse set of bioactive metabolites with distinct receptor affinities and biased signaling profiles, critically shaping itch, pain, and inflammatory responses in the skin. Disease‐associated alterations in protease expression favor the persistence of pruritogenic metabolites. Viewing substance P and its metabolites as a complex signaling system rather than a single mediator reveals novel therapeutic opportunities, including modulation of peptide metabolism, targeting receptor isoforms, and interference with alternative pathways such as MRGPRX2. Future antipruritic strategies will need to account for this complexity to achieve sustained clinical benefit.

## Ethics Statement

The authors have nothing to report.

## Consent

The authors have nothing to report.

## Conflicts of Interest

The authors declare no conflicts of interest.

## Data Availability

Data sharing not applicable to this article as no datasets were generated or analysed during the current study.
